# Acupuncture for Breast Cancer: A Systematic Review and Meta-Analysis of Patient-Reported Outcomes

**DOI:** 10.3389/fonc.2021.646315

**Published:** 2021-06-10

**Authors:** Yuzhu Zhang, Yang Sun, Dongmei Li, Xiaoyuan Liu, Chen Fang, Chunmin Yang, Tianyu Luo, Hai Lu, Huachao Li, Hongyan Zhang, Qianyi Liang, Jiahua Wu, Limei Huang, Rui Xu, Liping Ren, Qianjun Chen

**Affiliations:** ^1^ Breast Clinic Center, Guangdong Provincial Hospital of Chinese Medicine, Guangzhou, China; ^2^ Breast Clinic Center, The Second Affiliated Hospital of Guangzhou University of Chinese Medicine, Guangzhou, China; ^3^ National Resource Center for Chinese Materia Medica, China Academy of Chinese Medical Sciences, Beijing, China; ^4^ Breast Department, Zhuhai Hospital of Guangdong Province Hospital of Chinese Medicine, Zhuhai, China

**Keywords:** acupuncture, patient-reported outcome, breast cancer, systematic review, meta-analysis

## Abstract

**Abstract:**

The present systematic review and meta-analysis was undertaken to evaluate the effects of acupuncture in women with breast cancer (BC), focusing on patient-reported outcomes (PROs).

**Methods:**

A comprehensive literature search was carried out for randomized controlled trials (RCTs) reporting PROs in BC patients with treatment-related symptoms after undergoing acupuncture for at least four weeks. Literature screening, data extraction, and risk bias assessment were independently carried out by two researchers.

**Results:**

Out of the 2, 524 identified studies, 29 studies representing 33 articles were included in this meta-analysis. At the end of treatment (EOT), the acupuncture patients’ quality of life (QoL) was measured by the QLQ-C30 QoL subscale, the Functional Assessment of Cancer Therapy-Endocrine Symptoms (FACT-ES), the Functional Assessment of Cancer Therapy–General/Breast (FACT-G/B), and the Menopause-Specific Quality of Life Questionnaire (MENQOL), which depicted a significant improvement. The use of acupuncture in BC patients lead to a considerable reduction in the scores of all subscales of the Brief Pain Inventory-Short Form (BPI-SF) and Visual Analog Scale (VAS) measuring pain. Moreover, patients treated with acupuncture were more likely to experience improvements in hot flashes scores, fatigue, sleep disturbance, and anxiety compared to those in the control group, while the improvements in depression were comparable across both groups. Long-term follow-up results were similar to the EOT results.

**Conclusions:**

Current evidence suggests that acupuncture might improve BC treatment-related symptoms measured with PROs including QoL, pain, fatigue, hot flashes, sleep disturbance and anxiety. However, a number of included studies report limited amounts of certain subgroup settings, thus more rigorous, well-designed and larger RCTs are needed to confirm our results.

## Introduction

Globally, BC is the most prevalent cancer and the leading cause of cancer-related death in women. According to the International Agency for Research on Cancer (IARC), there are approximately 8 million BC survivors worldwide (1). The mean 5-year survival of women with BC (Of all cancer stages) is 85-90% in high-income countries (2, 3). The proportion of BC survivors continues to increase due to the growth and aging of the population as well as the development and improvement of early screening and treatment strategies (4). Although current therapeutic strategies are able to significantly improve the survival time, patients still suffer from treatment-related side effects of long-term therapy.

Patient-reported outcomes (PROs) refer to the treatment end results reported directly by patients (5, 6). Covering PROs such as the quality of life (QoL) allows for the incorporation of patients’ perspectives into the clinical evaluation, thus providing more accurate patient information and supporting joint decision making when developing new therapies (7). As a matter of fact, PROs become even more essential for BC patients in whom high survival rates are reached (8).

As a complementary and alternative medicine, the mechanism of action of acupuncture is not clear, but it is thought to be useful in improving complications such as lymphedema (9) and upper extremity impairment (10) caused by modified radical mastectomy, hot flashes (11, 12) and arthralgias (13) related to endocrine therapy, cancer-related fatigue (14), chemotherapy-related peripheral neuropathy (15) and the QoL (12), with less adverse effects. Meanwhile, some studies (16–19) found that acupuncture was not superior to standard management strategies for BC treatment-related complications. A number of systematic reviews on the application of acupuncture for the management of cancer treatment-related complications, most of which (20–22) focused on specific sequelae such as hot flashes and did not take PROs as main outcomes. An earlier comprehensive systematic review (23) of acupuncture in cancer care came up with the conclusion that the efficacy of acupuncture was not determined through its effects on symptoms such as pain, hot flashes, anxiety, or fatigue owing to the absence of the high-quality studies. The Society for Integrative Oncology (SIO) clinical practice guidelines (24) on the evidence-based use of integrative therapies during and after BC treatment recommended that acupuncture could be considered for anxiety, depression, fatigue, pain, hot flashes and QoL, but the use of acupuncture should be based on professional judgment and patient preferences due to the fact that the net benefit is small. Given the uncertainty about the efficacy of acupuncture in treating BC treatment-related symptoms, the purpose of this comprehensive systematic review and meta-analysis was to explore the benefits of acupuncture in patients with various BC treatment-related complications measured with PROs, thus providing more adequate evidence for the use of acupuncture in clinical practice.

## Methods

This work was performed according to the Preferred Reporting Items for Systematic Review and Meta-Analyses (PRISMA) guidelines (25). This study’s protocol registration number in PROSPERO is CRD42020199707.

### Search Strategy and Study Selection

The search for eligible articles was undertaken in three English databases (PubMed, Embase, Cochrane library) and four Chinese databases (CNKI, WanFan, Sinomed, VIP) from the launching to May 2020, and updated the literature to September, 2020. The medical subject headings and free text-word terms of “breast cancer” and “acupuncture” were used in combination as research terms. **Appendix S1** in the **Supplementary Materials** show the search strategy in the seven databases above. Furthermore, we checked the references listed in the included articles for additional qualifiers.

We included RCTs investigating the effect of acupuncture on complications associated with BC treatment in adult females (age>=18 years) who were diagnosed with BC of any tumor stage on pathology. These RCTs consisted of various acupuncture techniques with a needle, such as hand acupuncture and electroacupuncture, sham/placebo acupuncture, pharmacotherapy, no intervention or usual care as the controls, and reported the PROs. The exclusion criteria were as follows: 1) only included males; 2) patients enrolled with other cancers, or with ‘combination’ or ‘mixed’ cancers, but data on participants suffering from breast cancer-related sequelae could not be extracted; 3) enrolling patients with no breast cancer-related sequelae; 4) published as quasi-RCTs, cohort studies, case-control studies, cross-sectional studies, animal studies, case series and case reports; 5) not approved by an ethics committee.

The primary outcome was the QoL measured with validated scales such as the European Organization for the Research and Treatment of Cancer’s Core Quality of Life Questionnaire (EORTC QLQ-C30) and the FACT-G/B. The secondary outcomes were pain, hot flashes scores (frequency and severity of hot flashes), fatigue, sleep disturbances, depression and anxiety. The hot flashes score was defined as the product of the mean number of daily hot flashes and the mean daily severity.

Two reviewers (YZ.Z and Y.S) conducted the study selection independently, and a third reviewer (QJ.C or LP.R or R.X) was involved in the decision if there were discrepancies in the process.

### Data Abstraction

Two reviewers (YZ.Z and Y.S) independently abstracted the data recorded in the included studies using a standardized excel table. The following data were extracted in each eligible studies: authors, publication year, country, sample size, BC stage, age, sex, BC treatment-related sequelae, type of acupuncture, treatment protocol, controls, PROs (At the end of treatment and the extended follow up time), and the follow-up time. We abstracted data from the records for PROs of interest including the mean and standard deviation (continuous data), and the number of events (dichotomous data). If the primary studies merely reported median scores for the continuous data, we described them in the corresponding part in the review. If a study included both male and female BC, we only extracted data from females and obtained relevant information by contacting the authors if necessary. Data extraction was based on the intention-to-treat analysis. QJ.C or LP.R or R.X would take part in decision making in case YZ.Z and Y.S failed to reach an agreement on the differences in data extraction.

### Risk of Bias Assessment

The risks of bias in the included studies were assessed using the Cochrane Collaboration’s tool for assessing the risk of bias in randomized controlled trials (26). The tool covered 7 domains, and we considered that a high-quality study should have a low risk of bias in critical domains including the random sequence generation, blinding of participants and outcome assessment, incomplete outcome data, and selective reporting.

### Statistical Analysis

We calculated mean difference (MD) or standardized mean difference (SMD) and 95% confidence interval (CI) using the random-effect models for the mean change score of PROs measures in the acupuncture group and control group, and the Review Manager 5.3.5 software (27) was employed to conduct the meta-analysis. We selected the MD for PROs with the same measures, otherwise the SMD was chosen. We calculated the dichotomous data using the risk ratio (RR) with 95% CIs. The χ2 test and I2 statistics were used to explore the data’s heterogeneity. We changed the random-effect models to fixed-effect models in the data synthesis for sensitivity analysis. Besides, if a study provided both short-term and extended follow-up data for an outcome, we used the longest follow-up time data for the sensitivity analysis. We considered a P value of <0.05 to be statistically significant.

## Results

### Literature Search

**Figure 1:**
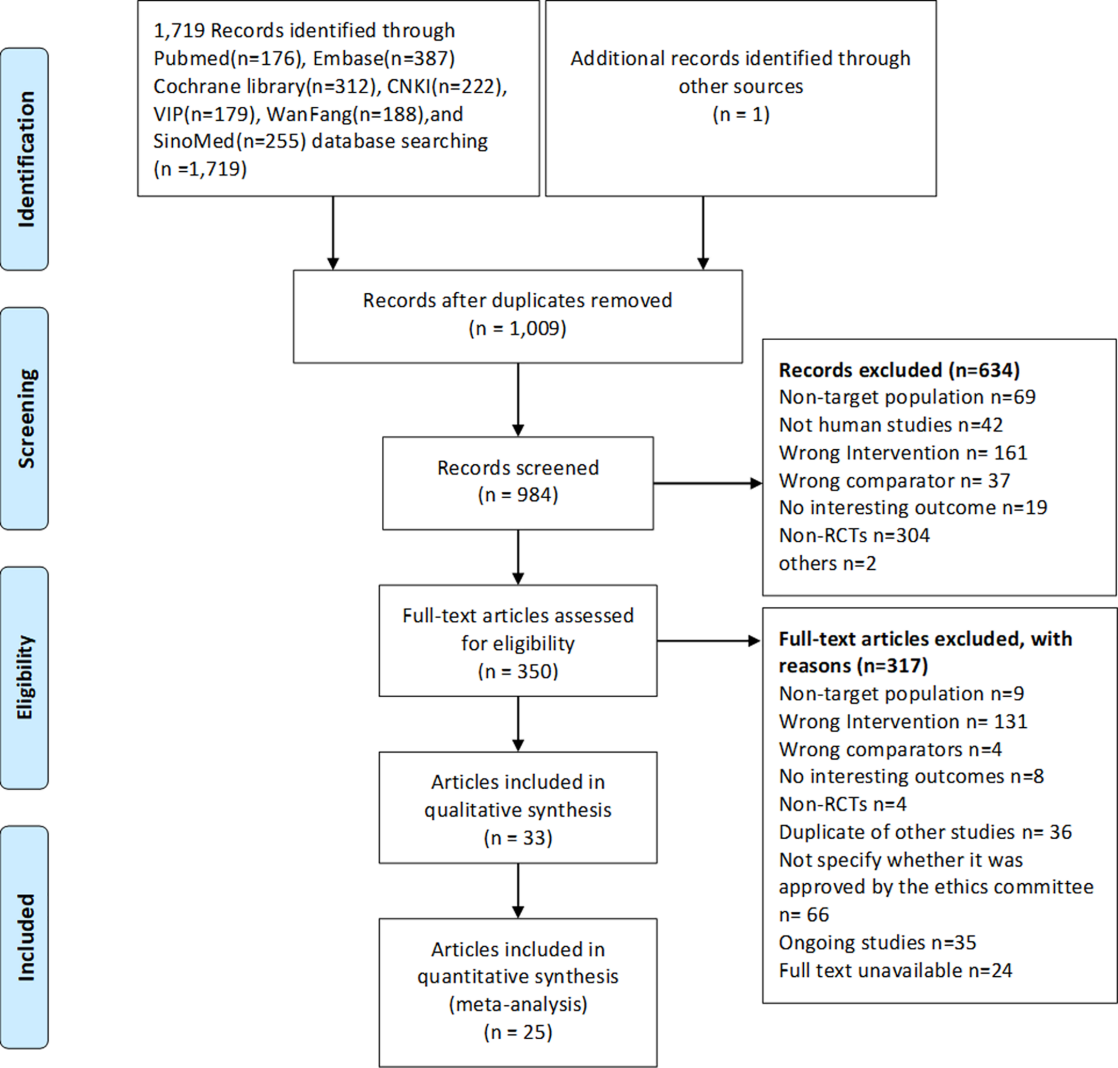
The PRISMA 2009 flow diagram for literature screening. RCTs randomized controlled trials.

Out of the 2,524 identified records, 184 potential articles were selected, and 33 articles (29 RCTs) (9–19, 28–49) published in English and Chinese between 2006 and 2020 were ultimately eligible. The PRISMA 2009 flow diagram for literature screening is illustrated in **Figure 1**.

### Characteristics and Risk Bias of the Eligible Studies

Of the 2,094 eligible patients in the 33 articles (9–19, 28–49), 1,091 patients received (electro)acupuncture and 1,003 received control strategies (sham (electro)acupuncture 474, no intervention 326, usual care 95, medicine 89, others 19). Twelve articles came from the USA, seven from China, three from Australia, three from Sweden, two from United Kingdom and the remaining seven articles were from seven different countries. Nine articles (10, 13, 16–18, 29, 36, 37, 45) were centered on musculoskeletal symptoms, seven (11, 12, 32, 43, 44, 47, 49) on hot flashes, five (14, 31, 38, 39, 41) on cancer-related fatigue, four (19, 40, 42, 48) on vasomotor symptoms, and the remaining six articles on peripheral neuropathy (15, 33), cognitive impairment (28, 30), chronic lymphedema (9), and physical and functional disorders (34), respectively. Patients enrolled in the eligible studies underwent acupuncture once or twice a week for 4 to 12 weeks, and each session lasted anywhere from 20 to 30 mins. The characteristics of the eligible studies are presented in **Appendix S2** in **Supplementary Materials**.

Approximately 90% of the articles specified the generation of random sequences, and 60% stated or used the allocation concealment. Therefore, selection bias was likely to be present in about a third of the included studies. Whereas roughly half of the studies might have a high risk in performance bias for lacking participants or outcomes assessment blinding. Among the qualified studies, most had low attrition and reporting biases. Within the 33 articles, 10 (11, 13, 31, 35–37, 40, 44, 45, 47) were judged as having low risks of bias in key domains, hence considered to be of high quality. The risks of bias assessment for all of the included articles were displayed in **Figures S1** and **S2** in **Supplementary Materials**.

### Outcome Measures

#### Quality of Life

A total of fourteen articles (9, 12–17, 19, 28, 30, 33, 41, 43, 45) comprising 1,225 patients reported the QoL, and used three generic and nine disease-specific QoL measures. At the end of treatment (EOT), the meta-analysis of three articles using generic QoL measures indicated that the QoL of patients in the acupuncture group improved significantly compared those in the control group. Bao, et al. declared that the median QoL of patients in the two groups were similar. For cancer-specific QoL measures, Oh et al. concluded that there were no significant differences in terms of the QoL measured by FACT-G between real and sham acupuncture, and the meta-analyses of two articles published by Crew et al. and Molassioti et al. indicated that the increase in the mean scores of three FACT-G/B domains for patients receiving acupuncture was significantly higher than that of patients in the control group. Comparison between the acupuncture group and the control group revealed that the increase in mean scores of the social/family well-being domain was almost statistically significant. Based on the standardized BC QoL measure FACT-B plus endocrine subscale (ES), i.e. FACT-ES, the patients treated with acupuncture also experienced an overall better QoL compared with those treated with the sham acupuncture or no intervention. Furthermore, three domains of the MENQOL including the physical score, vasomotor score and psychosocial score were decreased remarkably in the acupuncture group at the EOT. However, no statistically significant difference was detected between the acupuncture group and the control group with respect to the mean scores of the Functional Assessment of Cancer Therapy-Taxane (FACT-TAX) scales and neurotoxicity (FACT-NTX) subscales, FACT-COG QoL subscale, and Women’s Health Questionnaire (WHQ) at the EOT. The pooled analysis results of the changes in the mean scores of each scale were listed in **Table 1**.

**Table 1:** The QoL of acupuncture versus comparators for BC treatment-related symptoms.

Outcome or Subgroup	Participants	End of treatment		Extended follow-up time		Meaning of higher scores
		IV, Random, 95% CI	P value	IV, Random, 95% CI	P value	
QLQ-C30_quality of life subscale (15, 28)	154	MD 10.09 [7.26, 12.92]	P<0.0001^*^	MD 8.81 [5.77, 11.85]	P<0.0001^*^	better
Simplified and modified QLQ-C30 (9)	30	MD -1.10 [-1.25, -0.95]	P<0.0001^*^	NA	NA	worse
EuroQoL (EQ-5D) (16)	47	NA	P=0.14	NA	NA	better
FACT-G/B (17, 41, 45)						
Global (17)	32	NA	NS	NA	NA	better
Physical well-being (41, 45)	345	MD 4.40 [1.49, 7.30]	P=0.003^*^	NA	NA	better
Social/family well-being (41, 45)	345	MD 1.02 [0.01, 2.04]	P=0.05	NA	NA	better
Emotional well-being (41, 45)	345	MD 2.03 [1.11, 2.95]	P<0.0001^*^	NA	NA	better
Functional well-being (41, 45)	345	MD 3.49 [2.36, 4.62]	P<0.0001^*^	NA	NA	better
FACT-TAX (24)	63	MD -1.40 [-11.74, 8.94]	P=0.79	MD -4.50 [-16.99, 7.99]	P=0.48	better
FACT-NTX subscale (15, 24)	103	MD 4.40 [-1.58, 10.37]	P=0.15	MD -0.60 [-5.14, 3.94]	P=0.80	better
FACT-ES (13)	226	MD 3.26 [0.75, 5.77]	P=0.01^*^	MD 4.58 [1.85, 7.31]	P=0.001^*^	better
		RR 2.04 [1.30, 3.20]	P=0.002^*^	RR 1.88 [1.22, 2.90]	P=0.004^*^	better
FACT-COG QoL subscale (14, 30)	93	MD 1.95 [-0.69, 4.60]	P=0.15	NA	NA	better
MENQOL (12, 19)						
Global (19)	50	NA	NS	NA	NS	worse
Physical score (12)	190	MD -0.50 [-0.91, -0.09]	P=0.02^*^	MD -0.50 [-0.92, -0.08]	P=0.02^*^	worse
Sexual score (12)	190	MD -0.30 [-0.93, 0.33]	P=0.35	MD -0.55 [-1.17, 0.07]	P=0.08	worse
Vasomotor score (12)	190	MD -1.50 [-1.93, -1.07]	P<0.00001^*^	MD -1.38 [-1.88, -0.88]	P<0.00001^*^	worse
Psychosocial score (12)	190	MD -0.60 [-1.09, -0.11]	P=0.02^*^	MD -0.71 [-1.22, -0.20]	P=0.006^*^	worse
WHQ (43)	45	RR 1.52 [0.79, 2.94]	P=0.21	0.85 [0.51, 1.42]	P=0.53	better
PGWB (43)	45	NA	NA	NA	P=0.19	better

*P < 0.05. QLQ-C30, Quality-of-Life Questionnaire Core 30; EuroQol(EQ-5D), European quality-of-life survey; FACT-G/B, Functional Assessment of Cancer Therapy–General/Breast; FACT-TAX, The Functional Assessment of Cancer Therapy-Taxane (FACT-TAX); FACT-NTX subscale, Functional Assessment of Cancer Therapy-Neurotoxicity subscale; FACT-ES, Functional Assessment of Cancer Therapy-Endocrine Symptoms; FACT-COG, Functional Assessment Of Cancer Treatment Cognition Test; MENQOL, Menopause-Specific Quality of Life Questionnaire; WHQ, Women’s Health Questionnaire; PGWB, Psychological and General Well-being Index; NA, Not available; NS, Not significant; RR, Risk Ratio; MD, Mean Difference; IV, Inverse Variance; CI, Confidence Interval.

#### Pain

**Figure 2:**
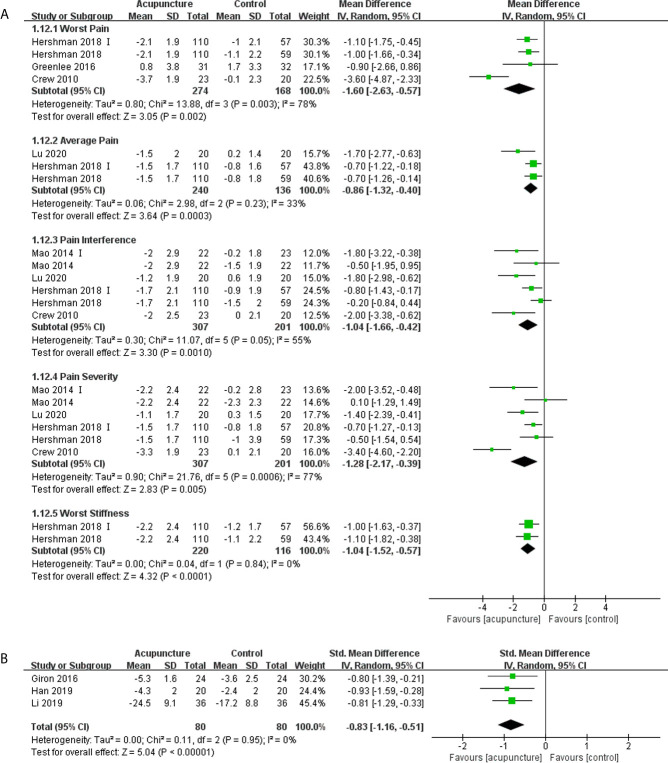
Forest plot of the change of pain in BC patients treated with acupuncture and control from the bassline to end of treatment. **(A)** Pain measured by Brief Pain Inventory-Short Form; **(B)** Pain measured by Visual Analogue Scale. IV, inverse variance; CI, Confidence Interval. The Roman numerals “I” followed the study ID represented the comparison of acupuncture versus no intervention in the study which had three arms.

Ten articles (10, 13, 15, 17, 18, 29, 33, 34, 37, 45) involving 712 patients reported the pain experienced by patients using the Brief Pain Inventory-Short Form (BPI-SF) or Visual Analog Scale (VAS), and higher scores were considered worse in all three patient-reported outcome measures (PROMs). Out of the ten articles, eight articles (10, 13, 15, 29, 33, 34, 37, 45) had available data that could be synthesized quantitatively. Treatment with acupuncture elicited a significant decline in the five subscales mean scores of the BPI-SF compared with control (13, 15, 33, 37, 45), and the meta-analyses results were presented in **Figure 2A**. In addition, the severity of pain in the acupuncture group also decreased significantly when compared with the control group as determined using the VAS measures (SMD -0.83, 95%CI, -1.16 to -0.51, P<0.00001, I^2 =^ 0) (**Figure 2B**) (10, 29, 34).

Oh et al. (17) found that there were no significant difference in BPI-SF pain severity and interference between the real and sham acupuncture group. Bao et al. (18) stated that the change in VAS at EOT results between the real (Median -2, range -68 to 53) and sham (Median -13, range -80 to 32) acupuncture groups was similar (P = 0.31).

#### Hot Flashes

**Figure 3:**
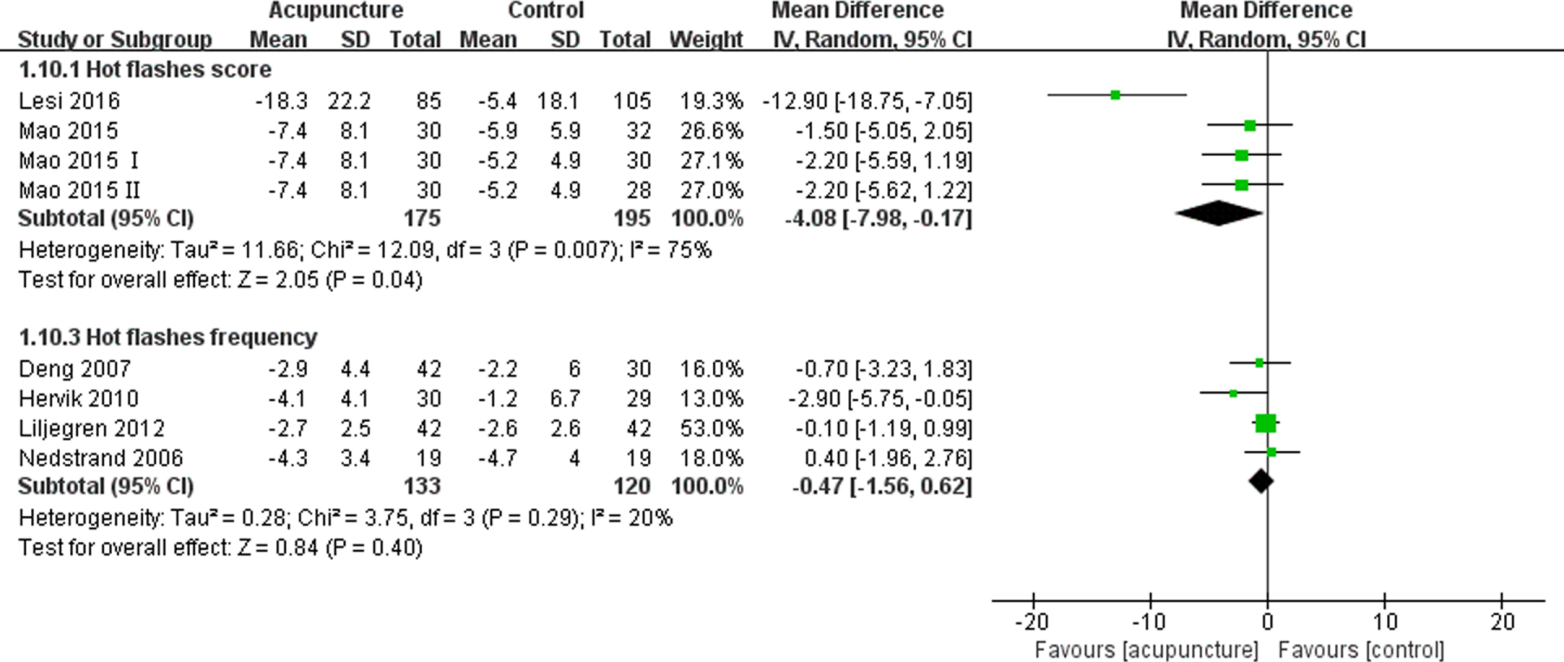
Forest plot of the change of hot flashes in BC patients treated with acupuncture and control from the bassline to end of treatment. IV, inverse variance; CI, Confidence Interval. The Roman numerals “I” and “II” followed the study ID represented the comparison of acupuncture versus no intervention and the comparison of acupuncture versus western medicine, respectively, in the study which had four arms.

Eleven articles (11, 12, 16, 19, 40, 42, 44, 46–49) enrolling 677 patients reported the patients’ daily hot flashes score or frequency, with six articles (11, 12, 42, 44, 47, 48) reporting data that could be quantitatively synthesized. The meta-analysis demonstrated that the difference in the hot flashes score were significant (MD -4.08, 95% CI, -7.98, -0.17, P= 0.04) (42, 44, 47, 48), but the difference in the hot flashes frequency (MD -0.47, 95% CI, -1.56, 0.62, P= 0.40) (11, 12) between the acupuncture and control groups were not significant (**Figure 3**). One study (40) concluded that more patients experienced a higher effect in terms of hot flashes in the acupuncture group compared with those in the sham group (RR 0.53,95% CI, 0.33, 0.88, P= 0.01).

Bao et al. (16) found that the median change in hot flashes weekly severity scores and frequency were similar between real and sham acupuncture groups. Meanwhile, the findings of another article (49) suggested that a remarkably greater percentage reduction in hot flashes severity was noted in BC patients who underwent acupuncture compared with those who received the placebo treatment (27.8% *vs.* 6.3%, P=0.017), and the decreased frequency of hot flashes was comparable between the two groups (47.4% *vs.* 23.7%, P=0.17). Walker et al. (19) discovered that both acupuncture and venlafaxine groups experienced similar changes in hot flash frequency and severity from the pre- to post-treatment period. The article published by Frisk et al. (46) did not provide any information on whether acupuncture was superior to hormone therapy, although patients’ hot flashes were significantly improved after treatment with acupuncture.

#### Fatigue

**Figure 4:**
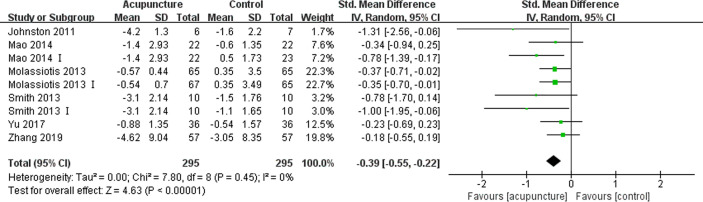
Forest plot of the change of fatigue in BC patients treated with acupuncture and control from the bassline to end of treatment. IV, inverse variance; CI, Confidence Interval. The Roman numerals “I” followed the study ID represented the comparison of acupuncture versus no intervention in the study which had three arms.

Six articles (14, 28, 31, 37–39) comprising 590 patients reported the fatigue experienced by patients using the Brief Fatigue Inventory (BFI), Multidimensional Fatigue Inventory-General Fatigue (MFI-GF) subscale, Piper fatigue scale (PFS) and QLQ-C30 fatigue subscale, with higher scores representing worse degrees of fatigue. The use of acupuncture significantly improved the fatigue of BC patients compared to those assigned to the control group (SMD -0.39, 95%CI, -0.55 to -0.22, P<0.00001, I2 = 0%) (**Figure 4**).

#### Sleep Disturbances

**Figure 5:**
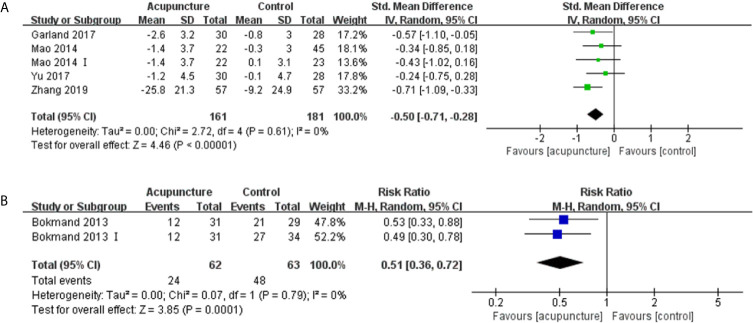
Forest plot of sleep disturbances in BC patients treated with acupuncture and control. **(A)** the changes in mean scores of sleep disturbances from the bassline to end of treatment; **(B)** Number of patients with sleep disturbance at the end of treatment. IV, inverse variance; CI, Confidence Interval. The Roman numerals “I” followed the study ID represented the comparison of acupuncture versus no intervention in the study which had three arms.

Five articles (16, 31, 32, 37, 40) with 371 patients reporting sleep disturbances using the Pittsburgh Sleep Quality Index (PSQI) and QLQ-C30 sleep disturbance subscale were identified, which the higher scores representing worse levels of sleep disturbances. Four of these articles (31, 32, 37, 40) had data that could be synthesized quantitatively. The patients receiving acupuncture experienced less sleep disturbances compared with those in the control group (SMD -0.50, 95%CI, -0.71 to -0.28, P<0.00001, I2 = 0%) (**Figure 5A**) (31, 32, 37). At the EOT, the acupuncture group had a lower number of patients suffering from sleep disturbances than the control group (RR 0.51, 95%CI, 0.36 to 0.72, P=0.0001, I2 = 0%) (**Figure 5B**) (40).

Bao et al. (16) found that the median change in PSQI scores were similar between the real and sham acupuncture groups.

#### Depression and Anxiety

**Figure 6:**
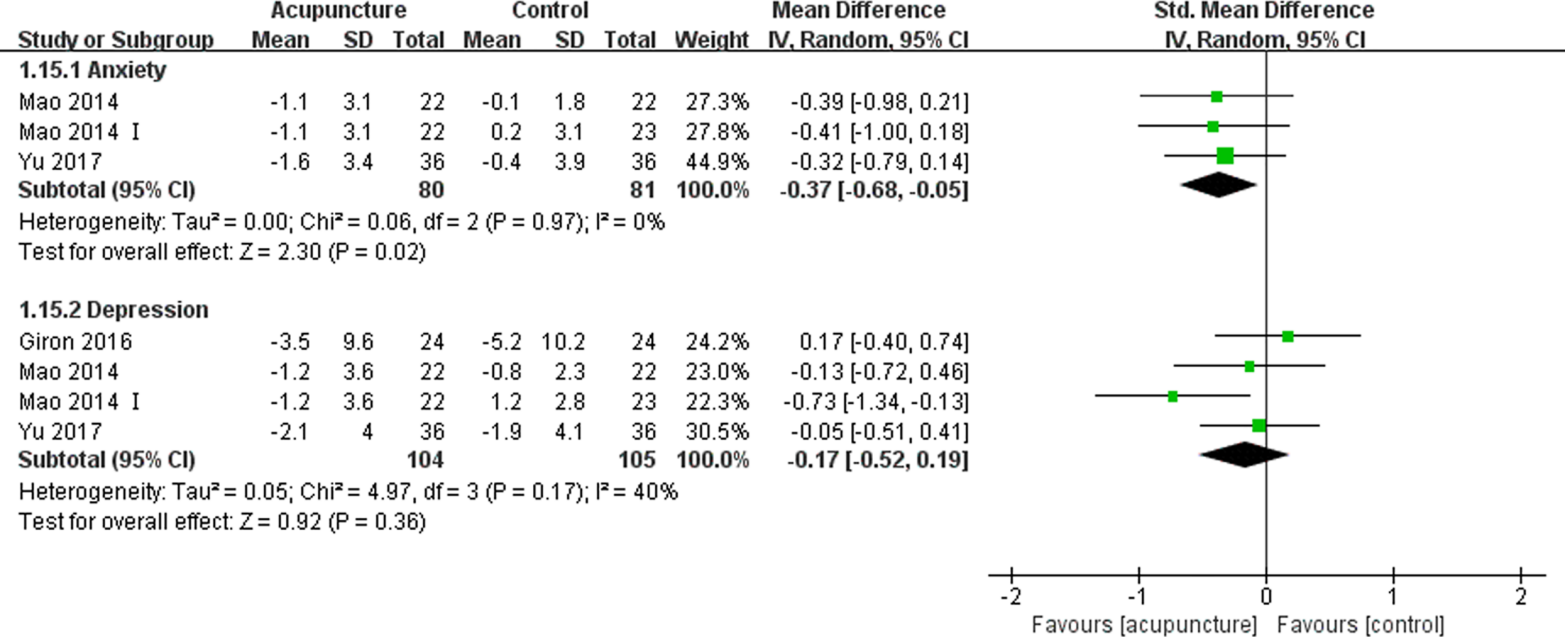
Forest plot of the change of anxiety and depression in BC patients treated with acupuncture and control from the bassline to end of treatment. IV, inverse variance; CI, Confidence Interval. The Roman numerals “I” followed the study ID represented the comparison of acupuncture versus no intervention in the study which had three arms.

Five articles (16, 19, 31, 37, 41) reported patients’ anxiety using the Hospital Anxiety and Depression Scale (HADS)-Anxiety subscale and Numeric Rating Scales (NRS) anxiety scale, in which higher scores indicated worse symptoms. A meta-analysis of two articles (31, 37) suggested that acupuncture could improve the anxiety of BC patients compared with the control management strategies (SMD -0.37, 95%CI, -0.68 to -0.05, P=0.02, I2 = 0%) (**Figure 6**). Six articles (16, 19, 31, 34, 37, 41) reported depression using the HADS-Depression subscale, Center for Epidemiological Studies Depression (CESD), and Beck questionnaire, in which higher scores suggest worse levels of depression. These articles did not report any significant difference between the acupuncture and control group (SMD -0.17, 95%CI, -0.52 to 0.19, P=0.36, I2 = 40%) (**Figure 6**) (31, 34, 37).

We conducted a descriptive analysis for the remaining articles describing the treatment-related depression and anxiety experienced by BC patients. Bao et al. (16) and Walker et al. (19) noticed similar changes in the severity of depression and anxiety between the acupuncture and control groups from the pre- to post-treatment period. Another article published by the Molassiotis et al. (41) drew the conclusion that acupuncture improved anxiety and depression using the HADS.

#### Adverse Effects

Twenty-two articles (9, 11–15, 17–19, 29, 31, 33, 35–40, 43, 45–47) mentioned adverse effects, and nine (9, 14, 18, 19, 29, 31, 39, 43, 46) of which clearly reported no adverse effect. Thirteen (11–13, 15, 17, 33, 35–38, 40, 45, 47) articles described specific adverse effects, such as pain or bleeding at the needle site, fatigue, pruritus, bruising, and dizziness, and these AEs were mild in severity. The remaining eleven articles (10, 16, 28, 30, 32, 34, 41, 42, 44, 48, 49) did not report any information on adverse effects.

#### Sensitivity Analysis

After changing the random-effects models to fixed-effects models, there was no significant change in the pooled analysis results of all predefined PROs. Additionally, we performed subgroup analyses of all the outcomes based on the different control strategies used, and the results are laid out in **Tables 2** and **3**. Furthermore, all of the high-quality studies were pooled to perform a sensitivity analysis, and the results indicated that the QoL of the patients receiving acupuncture was improved significantly, measured by the FACT-G physical well-being subscale, and there were more patients in the acupuncture group whose scores increased by more than 30% measured by the FACT-ES scale. The decrease in average pain score and worst stiffness score of BPI-SF in the acupuncture group were significantly different from those in the control group. The number of patients with improved sleep quality in the acupuncture group was significantly higher than in the control group. Additionally, acupuncture reduced mean scores on the scales measuring fatigue, and it almost reached a statistical significance. The pooled analysis results were detailed in **Table 4**.

**Table 2:** The effect of acupuncture on the QoL compared to different comparators.

Outcome or Subgroup	Participants	End of treatment		Meaning of higher scores
		IV, Random, 95% CI	P value	
**1. Acupuncture *vs.* sham acupuncture**				
QLQ-C30_quality of life subscale (28)	104	MD 10.10 [7.10, 13.10]	P<0.0001^*^	better
EuroQoL (EQ-5D) scale (16)	47	NA	P=0.14	better
FACT-G global scale (17)	32	NA	NS	better
FACT-G Physical well-being subscale (45)	43	MD 6.40 [2.93, 9.87]	P=0.0003^*^	better
FACT-G Social/family well-being subscale (45)	43	MD 0.70 [-2.86, 4.26]	P=0.70	better
FACT-G Emotional well-being subscale (45)	43	MD 3.00 [0.00, 6.00]	P=0.05	better
FACT-G Functional well-being subscale (45)	43	MD 2.80 [-0.70, 6.30]	P=0.12	better
FACT-TAX scale (33)	63	MD -1.40 [-11.74, 8.94]	P=0.79	better
FACT-NTX subscale (33)	63	MD 1.40 [-2.87, 5.67]	P=0.52	better
FACT-ES scale (13)	169	MD -3.38 [-6.92, 0.16]	P=0.06	better
FACT-ES scale^**^ (13)	169	RR 1.89 [1.03, 3.48]	P=0.04	better
**2. Acupuncture *vs.* no intervention**				
QLQ-C30_quality of life subscale (15)	40	MD 7.50 [2.94, 12.06]	P=0.001^*^	better
FACT-G Physical well-being subscale (41)	302	MD 3.30 [2.17, 4.43]	P< 0.001^*^	better
FACT-G Social/family well-being subscale (41)	302	MD 1.05 [-0.01, 2.11]	P=0.05	better
FACT-G Emotional well-being subscale (41)	302	MD 1.93 [0.96, 2.90]	P< 0.001^*^	better
FACT-G Functional well-being subscale (41)	302	MD 0.61 [2.38, 4.76]	P< 0.001^*^	better
FACT-ES scale (13)	167	MD -3.14 [6.69, 0.41]	P=0.08	better
FACT-ES scale^**^ (13)	167	RR 2.24 [1.14, 4.42]	P=0.02^*^	better
FACT-COG QoL subscale (14, 30)	93	MD 1.95 [-0.69, 4.60]	P=0.15	better
MENQOL Physical score (12)	190	MD -0.50 [-0.91, -0.09]	P=0.02^*^	worse
MENQOL Sexual score (12)	190	MD -0.30 [-0.93, 0.33]	P=0.35	worse
MENQOL Vasomotor score (12)	190	MD -1.50 [-1.93, -1.07]	P<0.00001^*^	worse
MENQOL Psychosocial score (12)	190	MD -0.60 [-1.09, -0.11]	P=0.02^*^	worse
**3. Acupuncture *vs.* western medicine**				
Simplified and modified QLQ-C30 (9)	30	MD -1.10 [-1.25, -0.95]	P<0.0001^*^	worse
MENQOL global scale (19)	50	NA	NS	worse
WHQ (43)	45	RR 1.52 [0.79, 2.94]	P=0.21	better
PGWB (43)	45	NA	NA	better

*P < 0.05.

**Proportion with >30% Improvement.

QLQ-C30, Quality-of-Life Questionnaire Core 30; EuroQol(EQ-5D), European quality-of-life survey; FACT-G/B, Functional Assessment of Cancer Therapy–General/Breast; FACT-TAX, The Functional Assessment of Cancer Therapy-Taxane (FACT-TAX); FACT-NTX subscale, Functional Assessment of Cancer Therapy-Neurotoxicity subscale; FACT-ES, Functional Assessment of Cancer Therapy-Endocrine Symptoms; FACT-COG, Functional Assessment Of Cancer Treatment Cognition Test; MENQOL, Menopause-Specific Quality of Life Questionnaire; WHQ, Women’s Health Questionnaire; PGWB, Psychological and General Well-being Index; NA, Not available; NS, Not significant; RR, Risk Ratio; MD, Mean Difference; IV, Inverse Variance; CI, Confidence Interval.

**Table 3:** The effect of acupuncture on the secondary outcomes compared to different comparators.

Outcome or Subgroup	Participants	End of treatment		Meaning of higher scores
		IV, Random, 95% CI	P value	
**1. Acupuncture *vs.* sham acupuncture**				
1.1 Pain				
BPI-SF worst pain subscale (13, 33, 45)	275	MD -1.84 [-3.59, -0.08]	P=0.04^*^	worse
BPI-SF average pain subscale (13)	169	MD -0.70 [-1.26, -0.14]	P=0.01^*^	worse
BPI-SF pain interference subscale (13, 36, 45)	256	MD -0.79 [-1.87, 0.28]	P=0.15	worse
BPI-SF pain severity subscale (13, 36, 45)	256	MD -1.28 [-3.35, 0.80]	P=0.23	worse
BPI-SF worst stiffness subscale (13)	169	MD -1.10 [-1.82, -0.38]	P=0.003^*^	worse
1.2 Hot flashes				
Hot flashes score (11)	62	MD -1.50 [-5.05, 2.05]	P=0.41	worse
Hot flashes frequency (42, 44, 47)	215	MD -0.81 [-2.30, 0.67]	P=0.28	worse
1.3 Fatigue (28, 31, 37, 38)	250	SMD-0.27 [-0.52, -0.02]	P=0.04^*^	worse
1.4 Sleep disturbances				
PSQI or QLQ-C30 sleep disturbance subscale (28, 31, 37)	239	SMD -0.47 [-0.78, -0.17]	P=0.002^*^	worse
No. of pts with sleep disturbance (40)	60	RR 0.53 [0.33, 0.88]	P=0.01^*^	worse
1.5 Anxiety (31, 37)	116	MD -1.09 [-2.21, 0.03]	P=0.06	worse
1.6 Depression (31, 37)	116	MD -0.30 [-1.60, 0.99]	P=0.64	worse
**2. Acupuncture *vs.* no intervention**				
2.1 Pain				
BPI-SF worst pain subscale (13)	167	MD -1.10 [-1.75, -0.45]	P=0.0009^*^	worse
BPI-SF average pain subscale (13, 15)	207	MD -1.09 [-2.04, -0.13]	P=0.03^*^	worse
BPI-SF pain interference subscale (13, 15, 36)	252	MD -1.27 [-2.01, -0.54]	P=0.0007^*^	worse
BPI-SF pain severity subscale (13, 15, 36)	252	MD -1.13 [-1.84, -0.43]	P=0.002^*^	worse
BPI-SF worst stiffness subscale (13)	167	MD -1.00 [-1.63, -0.37]	P=0.002^*^	worse
VAS (10, 29, 34)	160	SMD-0.83 [-1.16, -0.51]	P<0.00001^*^	worse
2.2 Hot flashes				
Hot flashes score (11, 12)	248	MD -7.28 [-17.75, 3.20]	P=0.17	worse
2.3 Fatigue (14, 35, 37, 39)	275	MD -0.50 [-0.76, -0.25]	P<0.0001^*^	worse
2.4 Sleep disturbances (37)	45	MD -1.50 [-3.50, 0.50]	P=0.14	worse
2.5 Anxiety (37)	45	MD -1.30 [-3.11, 0.51]	P=0.16	worse
2.6 Depression (34, 37)	93	MD -1.23 [-4.86, 2.39]	P=0.50	worse
**3. Acupuncture *vs.* western medicine**				
3.1 Hot flashes				
Hot flashes score (11)	60	MD -2.20 [-5.59, 1.19]	P=0.20	worse
3.2 Sleep disturbances (32)	58	MD -1.80 [-3.40, -0.20]	P=0.03*	worse
**4. Acupuncture *vs.* applied relaxation**				
4.1 Hot flashes				
Hot flashes frequency (48)	38	MD 0.40 [-1.96, 2.76]	P=0.74	worse

*P < 0.05.

**Proportion with >30% Improvement.

BPI-SF, Brief Pain Inventory-Short Form; VAS, Visual Analogue Scale; PSQI, Pittsburgh Sleep Quality Index; QLQ-C30, Quality-of-Life Questionnaire Core 30; RR, Risk Ratio; MD, Mean Difference; SMD, Standard Mean Difference; IV, Inverse Variance; CI, Confidence Interval.

**Table 4:** The pooled analysis results of high-quality articles for all predefined outcomes.

Outcome or Subgroup	Participants	End of treatment		Meaning of higher values
		IV, Random, 95% CI	P value	
**1. QoL**				
1.1. FACT-G				
Physical well-being (45)	43	MD 6.40 [2.93, 9.87]	P=0.0003^*^	better
Social/family well-being (45)	43	MD 0.70 [-2.86, 4.26]	P=0.70	better
Emotional well-being (45)	43	MD 3.00 [0.00, 6.00]	P=0.05	better
Functional well-being (45)	43	MD 2.80 [-0.70, 6.30]	P=0.12	better
1.2. FACT-ES (13)	169	MD -3.38 [-6.92, 0.16]	P=0.06	better
		RR 1.89 [1.03, 3.47] ^**^	P=0.04^*^	better
**2. Pain**				
2.1. BPI-SF				
Worst Pain (13, 45)	269	MD -2.24 [-4.79, 0.30]	P=0.08	worse
Average Pain (13)	226	MD -0.70 [-1.26, -0.14]	P=0.01^*^	worse
Pain Interference (13, 36, 45)	313	MD -0.79 [-1.87, 0.28]	P=0.15	worse
Pain Severity (13, 36, 45)	313	MD -1.28 [-3.35, 0.80]	P=0.23	worse
Worst Stiffness (13)	226	MD -1.10 [-1.82, -0.38]	P=0.003^*^	worse
**3. Hot flashes**				
3.1. Hot flashes score (11)	64	MD -1.50 [-5.05, 2.05]	P=0.41	worse
3.3.1 Hot flashes frequency (42, 44, 47)	215	MD -0.81 [-2.30, 0.67]	P=0.28	worse
**4. Fatigue**				
4.1 BFI or PFS (31, 37, 38)	136	SMD -0.34 [-0.68, -0.00]	P=0.05	worse
**5. Sleep disturbances**				
5.1. PSQI (31, 37)	125	MD -1.10 [-2.52, 0.32]	P=0.13	worse
5.2. No. of pts with sleep disturbance (40)	60	RR 0.53 [0.33, 0.88]	P=0.01^*^	worse
**6. Depression and anxiety**				
6.1. Anxiety (31, 37)	116	MD -0.35 [-0.72, 0.02]	P=0.23	worse
6.2. Depression (31, 37)	116	MD -0.08 [-0.44, 0.28]	P=0.67	worse

*p < 0.05.

**Proportion with >30% Improvement

FACT-G, Functional Assessment of Cancer Therapy–General; FACT-ES, Functional Assessment of Cancer Therapy-Endocrine Symptoms; BPI-SF, Brief Pain Inventory-Short Form; BFI, Brief Fatigue Inventory; PFS, Piper fatigue scale; PSQI, Pittsburgh Sleep Quality Index; pts, patients; NA, Not available; NS, Not significant; IV, Inverse Variance; CI, Confidence Interval.

Some studies reported results of the extended follow-up time after the end of treatment (EOT) with acupuncture. We performed a pooled analysis of every study reporting extended follow-up results, and the results of this analysis were presented in **Tables 1** and **5** for primary outcomes and secondary outcomes, respectively.

**Table 5:** The pooled analysis results of acupuncture versus comparators for BC treatment-related symptoms at extended follow up time.

Outcome	PROM	Participants	Estimate of the effect	P value	Meaning of higher scores
Pain	BPI-SF worst pain subscale (33)	63	MD 1.00, 95%CI [-0.28, 2.28]	P=0.13	worse
	BPI-SF pain interference subscale (37)	67	MD -1.44, 95%CI [-2.61, -0.26]	P=0.02^*^	worse
	BPI-SF pain severity subscale (37)	67	MD -1.42, 95%CI [-2.89, 0.05]	P=0.06	worse
Fatigue	BFI, PFS, QLQ-C30 fatigue subscale (28, 31, 37)	253	SMD -0.34, 95%CI [-0.57, -0.10]	P=0.006^*^	worse
Sleep disturbance	QLQ-C30 sleep disturbance subscale, PSQI (28, 37)	204	SMD -0.31, 95%CI [-0.77, 0.14]	P=0.18	worse
Anxiety	HADS-anxiety subscale (37)	67	MD -1.94, 95%CI [-3.14, -0.74]	P=0.002^*^	worse
Depression	HADS-depression subscale (37)	67	MD -0.80, 95%CI [-2.85, 1.26]	P=0.45	worse

*P < 0.05; BPI-SF, Brief Pain Inventory-Short Form; BFI, Brief Fatigue Inventory; PFS, Piper fatigue scale; QLQ-C30, Quality-of-Life Questionnaire Core 30; PSQI, Pittsburgh Sleep Quality Index; HADS, Hospital Anxiety and Depression Scale; RR, Risk Ratio; MD, Mean Difference; SMD, Standard Mean Difference; IV, Inverse Variance; CI, Confidence Interval.

## Discussion

This review pointed out that the use of acupuncture might improve the QoL, pain, hot flashes, fatigue, sleep disturbance and anxiety of BC patients compared with the use of control management strategies, while the difference in depression was not statistically significant between the two groups at EOT. At extended follow-up periods, improvements in the QoL, pain (pain interference domain), fatigue, and anxiety were also obvious. The subgroup analyses revealed that patients who underwent acupuncture appeared to experience more alleviation of sequelae than those who received no intervention, and similar to those who received a sham acupuncture or western medicine. The sensitivity analyses, which merely concentrated on high quality studies, suggested that the QoL (FACT-G physical well-being subscale and number of patients with a 30% improvement measured by FACT-ES), pain (average pain and worst stiffness subscale), and number of patients with sleep disturbances were ameliorated significantly.

Nowadays, PROs attract a tremendous deal of attention as healthcare keeps moving toward a more value-based framework to improve the quality of care. The PROs were measured on the basis of self-completed questionnaires called PROMs. The QoL or health-related QoL is a common PRO used as a primary or secondary outcome measure in cancer research, and it was more than 40 years ago that the first efforts to incorporate outcome measures into BC treatment trials were made (50). A variety of PROMs were used in the eligible studies enrolled in this review, not all of which favor treatment with acupuncture. We did not synthesize studies using different scales because of the heterogeneities in the constructs of each scale. For instance, some scales covering multiple dimensions were reported with only one total score, while others reported scores for each dimension separately. The subgroup analyses comparing acupuncture to different control management strategies suggested that there was a significant benefit in the QoL for patients who received acupuncture versus those who did not receive any intervention, irrespective of the PROMs used. Nonetheless, acupuncture elicited no obvious advantage in terms of improving patients’ QoL compared with sham acupuncture, measured by all the PROMs except the QLQ-C30 QoL and FACT-G physical well-being subscales. Only one study (9) revealed that compared with western medicine, acupuncture might improve the QoL of BC patients, but the author did not mention the reliability and validity of the measure used in the study. This finding was similar to those of a Cochrane systematic review (51) on hot flashes, which indicated that acupuncture lead to a significant improvement in the QoL compared to the control management strategies, and there was no statistically significant difference between the real acupuncture and sham acupuncture groups. The high-quality studies in which sham acupunctures were selected as controls suggested that the improvement in the QoL was obvious according to the FACT-G physical well-being subscale and FACT-ES. Despite the fact that we tried our best to conduct a subgroup analysis and a sensitivity analysis according to different control strategies and the quality of the eligible studies, inconsistent results were observed within each subgroup. Since the QoL results were extremely detailed (such the use of multidimensional PROMs) in some studies, identifying the key elements of inconsistency between different dimensions was quite challenging. The practitioner should identify appropriate patients depending on individual circumstances when providing treatment modalities.

Pain is a common symptom amongst patients with cancer, and a dozen of studies were published on the implementation of acupuncture for cancer pain. A recent systemic review (52) including 17 RCTs (With 1,111 patients) proved that acupuncture was closely associated with reduced cancer pain and decreased use of analgesics in comparison with sham acupuncture or wait-list controls. Likewise, this review also demonstrated that pain relief was obvious in patients receiving acupuncture compared with controls, and this improvement was multidimensional. Moreover, a subgroup analysis of this review determined that real acupuncture showed a significant improvement in all dimensions of pain compared with no intervention or wait-list controls, while only individual dimensions of the pain scale delineated any improvement compared with sham acupuncture.

A randomized trial (53) conducted in Australia that enrolled 327 patients with moderately severe menopausal hot flashes (Excluding BC patients) reported that acupuncture was not superior to noninsertive sham acupuncture in terms of symptoms alleviation. Subsequently, a systematic review (54) performed by the same team demonstrated that acupuncture is effective when compared with no treatment, but not efficacious compared with sham acupuncture. The pooled analysis of this review elucidated the fact that acupuncture might be useful in decreasing the hot flashes scores in the overall population, and the subgroup analysis and sensitive analysis did find positive results. This might be due to the small sample size or the inclusion of different target populations.

A previous systematic review (55) believed that acupuncture had a marked effect on fatigue in cancer patients, regardless of the comparison with sham acupuncture or standard care, particularly among breast cancer patients. Our review confirmed that the above findings, even the pooled analysis of the high quality-studies revealed the effect size was small.

The SIO clinical practice guidelines (24) declared that there was not enough evidence for the application of acupuncture in managing sleep disturbances. Even though some newly published studies (28, 31, 32) were supplemented to the pooled analysis, the significant improvement of sleep disturbance was not robust in the sensitivity analysis given that it was not completely consistent with the primary analysis. Acupuncture seemed to improve sleep disturbances compared to sham acupuncture based on the findings, but the limited number of rigorous studies prevented a convincing evidence from being obtained.

Relatively few studies had explored the effects of acupuncture for anxiety or depression in patients with cancer, and these syndromes were commonly used as secondary outcome measures in studies. Despite the fact that the SIO clinical practice guidelines (24) recommended that acupuncture could be considered for improving anxiety or depression, this review’s findings seemed to support that BC patients with anxiety merely benefit from acupuncture.

In this review, we established a comprehensive analysis for the PROs after the use of acupuncture to manage BC treatment-related symptoms. Although mixed results were observed in this review, we did find that acupuncture was associated with the improvement of BC treatment-related symptoms (11–13, 19, 41, 45, 47). Nevertheless, the effects of acupuncture in symptomatic relief were not obviously superior to those of the positive control management strategies including western medicine and sham acupuncture. We regarded sham acupuncture as an active control because it may exert positive physiological effects on patients (13). Moreover, through the sensitivity analysis, we noticed that the effects of acupuncture may persist for a long period of time after completing the intervention.

However, there were some inevitable limitations in the implementation process. First, since some PROs such as the QoL are often used as non-primary outcomes in studies, authors often do not present detailed results or even completely omit presenting them, which may lead to publication bias. Second, the included studies evaluating acupuncture reported mixed findings for some PROs, leading to a poor generalizability of these findings. Third, the small sample size of most of the included studies affected the interpretation of negative results. Notwithstanding, a lack of evidence does not necessarily translate to a lack of effect. Fourth, we found that the handling of missing data and QoL specific power calculations were frequently not mentioned in most eligible studies. Fifth, two-thirds of the eligible studies, which were not classified as high quality, were not blinded, but it is undeniable that a control without intervention could rule out natural history or regression of the symptoms.

## Conclusion

In a nutshell, acupuncture might improve BC treatment-related complications based on the measurements obtained from PROs including the QoL, pain, fatigue, hot flashes, sleep disturbance, and anxiety. The quality and quantity of the included studies limited the widespread use of acupuncture as the treatment of choice in clinical practice, and clinicians should provide patients with individualized treatment regimens based on specific factors (Such as patients’ preferences). Larger and better designed RCTs with long-term follow-ups are needed to confirm the efficacy of acupuncture.

## Data Availability Statement

The original contributions presented in the study are included in the article/**Supplementary Material**. Further inquiries can be directed to the corresponding authors.

## Author Contributions

All authors contributed to the article and approved the submitted version. QC proposed the conception and monitored the progress of the work. LR and RX designed the work and provided solutions to the inconsistencies. YZ, YS, and XL performed the literature search, study selection, data extraction, and wrote the manuscript. CF and CY undertook the data analysis. HLu, HLi, and HZ were responsible for the data interpretation. QL, JW, and LH helped to check the work. YZ and DL contributed to the revision of the manuscript. All the authors approved the submitted version, and agreed both to be personally accountable for the author’s own contributions and to ensure that questions related to the accuracy or integrity of any part of the work.

## Funding

This study was supported by grants from the National Natural Science Foundation of China (No. 81904206 and 81974571), Guangdong Natural Science Foundation (No. 2017A030313719), and Foundation Project of Guangzhou University of Traditional Chinese Medicine (No. XKP2019002).

## Conflict of Interest

The authors declare that the research was conducted in the absence of any commercial or financial relationships that could be construed as a potential conflict of interest.
