# Supplementary material for: Acupuncture for Breast Cancer: A Systematic Review and Meta-Analysis of Patient-Reported Outcomes
Source: Front Oncol. 2021 Jun 10;11:646315. doi: 10.3389/fonc.2021.646315 (PMC8222976; doi:10.3389/fonc.2021.646315)

Supplementary Materials

**Supplementary materials caption**

Appendix S1 Literature search strategies

Appendix S2 The basic characteristic of the eligible studies

Figure S1 Risk of bias graph, “Green” represents low risk of bias, “Red” represents high risk of bias, and “Yellow” represents unclear risk of bias.

Figure S2 Risk of bias summary, “Green” represents low risk of bias, “Red” represents high risk of bias, and “Yellow” represents unclear risk of bias.

Appendix 1 Literature search strategies

**PubMed**

((("Breast Neoplasms"[Mesh]) OR ((Mammary OR breast) AND (cancer OR tumor OR carcinoma OR neoplasm))) AND ((((("Acupuncture"[Mesh] OR "Acupuncture Therapy"[Mesh]) OR "Auriculotherapy"[Mesh]) OR "Cupping Therapy"[Mesh]) OR ("Dry Needling"[Mesh])) OR ((acupuncture OR electroacupuncture OR acupressure OR auriculotherapy OR moxibustion OR cupping) OR (dry needling)) AND (random OR randomization OR randomized OR randomised OR randomly)

**Cochrane Library**

#1 MeSH descriptor: [Breast Neoplasms] explode all trees

#2 Mammary OR breast

#3 cancer OR tumor OR carcinoma OR neoplasm

#4 #2 AND #3

#5 #1 OR #4

#6 MeSH descriptor: [Acupuncture] explode all trees

#7 MeSH descriptor: [Acupuncture Therapy] explode all trees

#8 MeSH descriptor: [Auriculotherapy] explode all trees

#9 MeSH descriptor: [Cupping Therapy] explode all trees

#10 MeSH descriptor: [Dry Needling] explode all trees

#11 (acupuncture OR electroacupuncture OR acupressure OR auriculotherapy OR moxibustion OR cupping) OR (dry needling)

#12 #6 OR #7 OR #8 OR #9 OR #10 OR #11

#13 random OR randomization OR randomized OR randomised OR randomly

#14 #5 AND #12 AND #13 in Trials

**EMBASE**

| No. | Query |
| --- | --- |
| #25 | #11 AND #18 AND #24 |
| #24 | #19 OR #20 OR #21 OR #22 OR #23 |
| #23 | randomly' |
| #22 | randomised' |
| #21 | randomized' |
| #20 | randomization' |
| #19 | random' |
| #18 | #12 OR #17 |
| #17 | #13 OR #14 OR #15 OR #16 |
| #16 | auriculotherapy':ab,kw,ti |
| #15 | acupressure':ab,kw,ti |
| #14 | electroacupuncture':ab,kw,ti |
| #13 | acupuncture':ab,kw,ti |
| #12 | 'acupuncture'/exp |
| #11 | #1 OR #10 |
| #10 | #4 AND #9 |
| #9 | #5 OR #6 OR #7 OR #8 |
| #8 | carcinoma':ab,kw,ti |
| #7 | neoplasm':ab,kw,ti |
| #6 | cancer':ab,kw,ti |
| #5 | tumor':ab,kw,ti |
| #4 | #2 OR #3 |
| #3 | mammary':ab,kw,ti |
| #2 | breast':ab,kw,ti |
| #1 | breast tumor'/exp |

**China Academic Journal Network (CNKI)**

(SU=’乳腺癌’ OR SU=’乳腺肿瘤’) AND (SU=’针灸’ OR SU=’艾灸’ OR SU=’头针’ OR SU=’耳针’ OR SU=’三棱针’ OR SU=’拔罐’ OR SU=’穴位注射’ OR SU=’皮肤针’ OR SU=’皮内针’ OR SU=’穴位敷贴’ OR SU=’穴位埋线’ OR SU=’电针’ OR SU=’耳穴’ OR SU=’针’ OR SU=’穴’ OR SU=’刺’) AND FT='随机

**WanFang Data**

(主题:"乳腺癌" OR 主题:"乳腺肿瘤") AND (主题:"针灸" OR 主题:"艾灸" OR 主题:"头针" OR 主题:"耳针" OR 主题:"三棱针" OR 主题:"拔罐" OR 主题:"穴位注射" OR 主题:"皮肤针" OR 主题:"皮内针" OR 主题:"穴位敷贴" OR 主题:"穴位埋线" OR 主题:"电针" OR 主题:"耳穴") AND (全部:"随机")

**Chinese science and technology journals database (VIP)**

M=(乳腺癌 OR 乳腺肿瘤) AND M=(针灸 OR 艾灸 OR 头针 OR 耳针 OR 三棱针 OR 拔罐 OR 穴位注射 OR 皮肤针 OR 皮内针 OR 穴位敷贴 OR 穴位埋线 OR 电针 OR 耳穴)

**Chinese biomedical literature service system (SinoMed)**

1# "针灸疗法"[不加权:扩展]

2# 针灸 OR 艾灸 OR 头针 OR 耳针 OR 三棱针 OR 拔罐 OR 穴位注射 OR 皮肤针 OR 皮内针 OR 穴位敷贴 OR 穴位埋线 OR 电针 OR 耳穴

3# "乳腺肿瘤"[不加权:扩展]

4# 乳腺癌 OR 乳腺肿瘤

5# 随机

6# (针灸 OR 艾灸 OR 头针 OR 耳针 OR 三棱针 OR 拔罐 OR 穴位注射 OR 皮肤针 OR 皮内针 OR 穴位敷贴 OR 穴位埋线 OR 电针 OR 耳穴) OR ("针灸疗法"[不加权:扩展])

7# (乳腺癌 OR 乳腺肿瘤) OR ("乳腺肿瘤"[不加权:扩展]) 485144 2020-06-02 10:04:09.0

8# ((乳腺癌 OR 乳腺肿瘤) OR ("乳腺肿瘤"[不加权:扩展])) AND ((针灸 OR 艾灸 OR 头针 OR 耳针 OR 三棱针 OR 拔罐 OR 穴位注射 OR 皮肤针 OR 皮内针 OR 穴位敷贴 OR 穴位埋线 OR 电针 OR 耳穴) OR ("针灸疗法"[不加权:扩展]))

9# (((乳腺癌 OR 乳腺肿瘤) OR ("乳腺肿瘤"[不加权:扩展])) AND ((针灸 OR 艾灸 OR 头针 OR 耳针 OR 三棱针 OR 拔罐 OR 穴位注射 OR 皮肤针 OR 皮内针 OR 穴位敷贴 OR 穴位埋线 OR 电针 OR 耳穴) OR ("针灸疗法"[不加权:扩展]))) AND (随机)

**Appendix S2 The basic characteristic of the eligible studies**

| **Study ID** | **Year** | **Country** | **Study period** | **Population** | **Stage** | **Sample size** | **Age*** | **Intervention** | **Acupuncture points** | **Needle retaining time** | **Acupuncture regimen** | **Follow-up time** |
| --- | --- | --- | --- | --- | --- | --- | --- | --- | --- | --- | --- | --- |
| Lu[15] | 2020 | Korea | Jun 2014 to Jul 2016 | Peripheral Neuropathy | I-III | A: 20 C: 20 | A: 54.0 (32.0 to 68.0) C: 53.5 (26.0 to 71.0) | A: acupuncture +electroacupuncture C: usual care | Primary points: Yin Tang, SP-9, ST-36, SP-6, K-3, LR-3, Qiduan(1st-5th), L1-11, TW-5, Baxie | 30 min | 18 sessions over 8 weeks | 8 weeks |
| Zhang[28] | 2019 | China | Octr 2015 to Dec 2017 | Cognitive impairment | Ⅰ-Ⅳ | A: 57 C: 57 | A: 45.08±5.97 C: 45.61±5.08 | A: acupuncture C: non-insertive stimulation at non-acupuncture points | St-36 (bilateral), SP-10, CV-17, CV-12, GV-20 CV-7, GV-16, Bl-15 (bilateral), Bl-45 (bilateral), HT-5 (bilateral), KI-6 (bilateral) | 20 min | twice a week for 8 weeks | 8/16 weeks |
| Li[29] | 2019 | China | Jul 2016 to Sep 2017 | Muscle, bone and joint pain | Ⅰ-Ⅲ | A: 36 C: 36 | A: 57.78±7.54 C:58.21±7.83 | A: acupuncture C: no intervention | Ashi point | 20 min | 5 days per week for 3 months | 12 weeks |
| Han[10] | 2019 | China | Mar 2017 to Dec 2018 | Shoulder joint dysfunction | NA | A: 20 C: 20 | A: 55.60±6.90 C: 53.80±7.52 | A: acupuncture C: no intervention | Ashi point | NA | twice a week for 12 weeks | 12 weeks |
| Tong[30] | 2018 | China | May 2017 to Oct 2017 | Cognitive Impairment | Ⅰ-Ⅱ | A: 40 C: 40 | A: 43.11±4.23 C: 42.26±4.42 | A: acupuncture C: no intervention | Primary points: DU20, EX-HN1, KI3; Secondary points: DU-24, K14, GB41, ST-36 | 30 min | once a day for 5 days +2 days of rest for 8 weeks | 8 weeks |
| Hershman[13] | 2018 | USA | Mar 2012 to Feb 2017 | Joint pain | I-III | A: 110 C1: 59 C2: 57 | A: 60.8 (34.1 to 80.6) C1: 57.0 (40.6 to 77.5) C2: 60.6 (27.1 to 76.0) | A: acupuncture C1: sham acupuncture C2: waitlist control | Full body points SJ 5, LI 4, GB 41, GB 34,ST 41,KD 3; Auricular points Shen Men, Kidney, Liver, Upper Lung, Sympathetic; Shoulder LI 15, SJ 14,SI 10; Wrist SI 5,SJ 4,LI5; Fingers SI 3,Ba Xie,LI 3; Lumbar DU 3, DU 8,UB23; Hip GB 30, GB 39; Knee SP 9,SP 10,St 34 | 20 to 25min | 2 sessions per week for 6 weeks + 1 session per week for 6 weeks | 12 weeks |
| Yu[31] | 2017 | China | Jan 2013 to Jun 2014 | Fatigue | I-III | A: 36 C: 36 | A: 50.2±8.0 C: 51.4±8.4 | A: acupuncture C: non-insertive stimulation at non-acupuncture points | GV 20, PC6, CV 6, St 36, SP-6 | NA | twice a week for 4 weeks | 4/8 weeks |
| Garland [33] | 2017 | USA | Nov 2009 to Jun 2013 | HF | I-III | A: 30 C: 28 | A: 52.9±8.6 C: 50.4±8.4 | A: electro-acupuncture C: gabapentin | Primary points**:  Supine: Kd 3 (bi), Sp 6 (bi), Ren 4;  Prone: Kd 3 (bi), Sp 6 (bi), UB 23 | 30 minutes | twice a week for 2 weeks + once weekly for 6 more weeks | 8 weeks |
| Lesi[12] | 2016 | Italy | Mar 2010 to Oct 2013 | HF | NA | A: 85  C: 105 | A: 50(27 to 63)C: 49(37 to 65) | A: acupuncture  C: no intervention | Three common acupoints (SP 6, LI 11, CV 4) plus the following point according to different syndrome:  Kidney yin emptiness: LU7, KI6, KI3, KI10, HE6, KI7; Kidney yin and yang deficiency KI3, LU7, KI6, HE6, BL52, KI7,  ST36, CV6, GV20, BL23; Kidney and liver yin and yang deficiency and yang escape from liver: LR3, LI4, GB20, KI6, PC7,  LU7, LR2, ST37; Kidney and heart  Disharmony: KI6, LU7, CV14, KI2, PC6, KI3, KI13, HT6, KI7, HT8, LU7, CV15, DU24, ST37; Phlegm or qi stasis: CV17, PC6, SP9, SP10, ST40, LU7, KI6, CV6, CV10, TE6, ST28; Blood stasis: SP4, PC6, KI14, SP10, BL17, CV4, CV6, LR3, LU7 | 20 minutes | once per week for 12 weeks | 12weeks/3/6months |
| Greenlee[33] | 2016 | USA | Feb 2011 to Oct 2014 | Peripheral neuropathy | I-III | A: 31 C: 32 | A: 51.8±10.7 C: 48.3±12.0 | A: electroacupuncture C: sham electroacupuncture | General GB34, ST36, LI4, LI10; lower limb L3, L5; upper limb C5, C7 | 30 min | 2 days a week for 12 weeks | 6/12/16weeks |
| Giron[34] | 2016 | Brazil | NA | Physical and functional disorders | NA | A: 24 C: 24 | 53.7 ± 11.1 | A: acupuncture C: no intervention | CV 3, SP 9, ST 36, KI 7, LR 3, GB 21, LI 15, HT 14, 5 LU, LI 4, ST 38, BL 60 | 30 min | once per week 10 weeks | 1/5/10 weeks |
| Yao[9] | 2016 | China | Jan to Dec 2013 | Chronic lymphedema | NA | A: 15  C: 15 | A: 56.2±5.82  C: 55.8±5.02 | A: warm acupuncture  C: diosmin | LI 10, LI 11, LI 14, LI 15, SJ 5, SJ 14 | 30 min | on alternate days for a period of 30 days | 4 weeks |
| Mao[11] | 2015 | USA | Nov 2009 to Jun 2013 | HF | I-III | A1: 30 C1: 32 C2: 28 C3: 30 | A1: 52.9 ± 8.6 C1: 52 ± 8.9 C2: 50.4 ± 8.4 C3: 52.6 ± 8.2 | A1: electroacupuncture C1: sham electroacupuncture C2: gabapentin C3: Placebo | Primary points**:  Supine: Kd 3 (bi), Sp 6 (bi), Ren 4;  Prone: Kd 3 (bi), Sp 6 (bi), UB 23 | 30 min | twice per week for 2 weeks+ once per week for 6 more weeks | 8/12/24weeks |
| Mao[36,37] | 2014 | USA | Sep 2009 to May 2012 | Arthralgia | I–III | A1: 22 C1: 22 C2: 23 | A1: 57.5 ± 10.1 C1: 60.9 ± 6.5 C2: 60.6 ± 8.2 | A1: electroacupuncture C1: sham electroacupuncture C2: waitlist control | Shoulder LI15, SJ14, SI9, SI10; Scapula SI11, SI12, SI14, UB43; ElbowLI11, Lu5, SJ10, SJ5, LI4); Hand/Finger SI3, LI3, Extra; Hip GB30, UB37, GB29; Knee St.34, St. 35, Extra, GB34, GB 35, Sp 9, UB 57, UB 58); Ankle St.41, Sp. 5, GB40, UB60, K3; Foot/Toe Sp.4, UB65, Extra | 30 min | twice per week for 2 weeks + once per week for 6 more weeks | 4/8/12weeks |
| Bao[16] | 2014 | USA | May 2008 to Jul 2011 | Musculoskeletal Symptoms | I–III | A: 25 C: 26 | A: 61 (44 to 82) C: 61 (45 to 85) | A: acupuncture C: sham acupuncture | CV 4, CV6, CV12, bilateral LI 4, MH 6, GB 34, ST 36, KI 3, BL 65 | 20min | once week for 8 weeks | 4/8/12weeks |
| Bao[18] | 2013 | USA | May 2008 to Jul 2011 | Musculoskeletal symptoms | I–III | A: 25 C: 26 | A: 61 (44 to 82) C: 61 (45 to 85) | A: acupuncture C: sham acupuncture | CV 4, CV6, CV12, bilateral LI 4, MH 6, GB 34, ST 36, KI 3, BL 65 | 20min | once week for 8 weeks | 4/8/12weeks |
| Smith[35,38] | 2013 | Australia | Apr 2010 to Feb 2011 | Fatigue and well-being | NA | A: 10 C1: 10 C2: 10 | A: 55.0±8.8 C1: 53.0±12.5 C2: 58.0±7.5 | A: acupuncture C1: sham acupuncture C2: waitlist control | Primary points: bilateral KI3, KI27, ST36, SP6, unilateral CV4, CV6;  Secondary points: Not report | 20 to 45min | twice weekly over 3 weeks + once weekly for 3 weeks | 2/4/6 weeks |
| Oh[17] | 2013 | Australia | Jun 2009 to Aug 2011 | Arthralgia | I, II or IIIa | A: 16C:16 | NA | A: electroacupuncture  C: sham electroacupuncture | Day 1***:LI4, LI11, GB34, ST40, LR3, GV20, Shishencong and Baxie;  Day 2***: GB21, TE5, ST36, SP6, LR3, GV20, Shishencong and Baxie | 20 min | twice weekly for 6 weeks | 6/12 weeks |
| Molassiotis[41] | 2012 | United Kingdom | Jun 2009 to August 2011 | Fatigue | I, II or IIIa | A: 227 C:75 | A: 52 (30 to 75) C: 53(25 to 80) | A: acupuncture C: enhanced usual care | Primary points: bilaterally or unilaterally three points (ST36, SP6, and LI4);  Alternative points: GB34, SP9 | 20min | once a week for 6 weeks | 6 weeks |
| Molassiotis[39] | 2013 | United Kingdom | Jun 2009 and Aug 2011 | Fatigue | I, II or IIIa | A1: 67 A2: 65 C2: 65 | NA | A1: self-acupuncture A2: acupuncture C2: no maintainance | Primary points: bilaterally or unilaterally ST36, SP6, and LI4;  Alternative points: GB34, SP9 | 20min | once a week for 4 weeks |  |
| Bokmand[40] | 2013 | Denmark | NA | Menopausal discomfort | NA | A: 31 C1: 29 C2: 34 | A: 60 (46 to 75) C1: 62 (43 to 72) C2: 62 (45 to 72) | A: acupuncture C1: sham acupuncture C2: no treatment | HC6, KI3, SP6, LR3 | 15 to 20 min | once a week for 5 weeks | 5/11/17weeks |
| Liljegren[42] | 2012 | Sweden | NA | Vasomotor symptoms | NA | A: 42 C: 42 | A: 58±6.8 C: 58±9.3 | A: acupuncture C: non-insertive stimulation at non-acupuncture points | Unilaterally Li4, Ht6, LR3, St36 and bilaterally Sp6, Ki7 | 20 min | twice weekly for 5 weeks | 3/6/18 weeks |
| Frisk[43] | 2012 | Sweden | NA | HF | NA | A: 27 C: 18 | A: 54.1 (47 to 69) C: 53.4 (43 to 67) | A: electroacupuncture C: hormone therapy | Bilaterally UB 15, UB 23, UB 32 and unilaterally GV 20, H 7, P 6, LIV 3, SP 6, SP 9 | 30 min | twice a week for 2 weeks+ once a week for 10 weeks | 3/6 /9/12/18 months |
| Frisk[46] | 2008 | Sweden | NA | HF | NA | A: 27 C: 18 | A: 54.1 (47–69) C: 53.4 (43-67) | A: electroacupuncture C: hormone therapy | Bilaterally UB 15, UB 23, UB 32 and unilaterally GV 20, H 7, P 6, LIV 3, SP 6, SP 9 | 30 min | twice a week for 2 weeks+ once a week for 10 weeks | 3/6 /9/12/18/24 months |
| Johnston[14] | 2011 | USA | NA | Fatigue | NA | A: 6 C: 7 | A: 55.0±6.4 C: 53.0±7.2 | A: acupuncture C: no treatment | Primary points: Li4, Sp6, ST36, Ki3  secondary points: gastrointestinal symptom P6, Sp4; emotional symptom Li4, Sp6, ST36, Ki3, ,Lu7, Ki4, Liver 3, Yintang, Gv20; sleep symptom H7, Ki4, and Ub62, Gb20, Te5, Gb43, SI3, Ub62; pain symptom Gb29, Gb30, Gb40 | 30 min | once a week for 8 weeks | 8 weeks |
| Walker[19] | 2010 | USA | Sep 2004 to Jun 2007 | Vasomotor symptoms | 0-III | A: 25 C: 25 | A: 52.2±10.3 C: 56.6±8.3 | A: acupuncture C: venlafaxine | Primary points: urinary bladder 23, kidney 3, and spleen 6;  Secondary points: du 14, gallbladder 20, lung 9, liver 3, du 20, stomach 36, ren 6, pericardium 7, heart 7 | 30 min | twice a week for 4 weeks + once per week for 8 weeks | 3/6/9/12 months |
| Hervik[44] | 2010 | Norway | Mar 2003 to Dec 2006 | HF | NA | A: 30 C: 29 | A: 53.6 ± 6.4 C: 52.3 ± 6.9 | A: acupuncture C: sham acupuncture | LIV3, GB20, LU7, KI3, SP6, REN4, P7, LIV8 | 30 min | twice weekly for 5 weeks+ once a week for 5 weeks | 10/12 weeks |
| Crew[45] | 2010 | Taiwan | May 2006 to Sep 2008 | Joint symptoms | I to III | A: 23C: 20 | A: 58 (44 to 77)C: 57 (37 to 77) | A: acupuncture  C: sham acupuncture | Full-body points TB 5, GB 41-zulin qi, GB 34-yang ling quan, LI 4-he gu, ST-41-jie xi, KD 3-tai xi; auricular points shen men, kidney, liver, upper lung, and sympathetic; the joint-specific point protocols are as follows: knee SP-9, SP-10, ST-34; fingers SI-5, SI3, ba xie, LI-3; lumbar Du-3, Du-8, UB-23; shoulder LI-15, SJ-14, SI-10; hip GB-30, GB-39, wrist SJ-4, LI-5. | 20 to 25 minutes | 12 sessions over 6 weeks | 3/6 weeks |
| Deng[47] | 2007 | USA | Nov 2002 to Dec 2005 | HF | NA | A: 42 C: 30 | A: 55(48 to 77) C: 56(49 to 59) | A: acupuncture C: sham acupuncture | DU 14, GB 20, BL 13, PC 7, H 6, K 7, ST 36, SP 6, ear shen men, ear sympathetic point | 20 min | twice weekly for 4 weeks | 4/6/12/24weeks |
| Nedstrand[48] | 2006 | Sweden | NA | Vasomotor symptom | NA | A: 19 C: 19 | 53 (30 to 64) | A: electro-acupuncture C: applied relaxation | BL15,23 and 32 bilaterally (paraspinally at thoracic and lumbar levels), HT 7 (wrist), SP 6 and 9 (lower leg), LR 3 (foot), PC 6 (wrist), and GV 20 (head) | 30min | twice a week for 2 weeks +once a week for 10 weeks | 4/6/12/24weeks |
| Huang[49] | 2006 | USA | Mar 2003 to Jan 2004 | HF and sleep quality | NA | A: 12 C: 17 | A: 56.92±1.73 C: 53.71± 4.24 | A: active acupuncture C: placebo acupuncture | Primary points**:  Supine: Kd 3 (bi), Sp 6 (bi), Ren 4;  Prone: Kd 3 (bi), Sp 6 (bi), UB 23; | 20 min | twice a week for 2 weeks +once a week for 5 weeks | 7 weeks |

*mean±standard deviation or median (interquartile range) or median (range); ** Please referred to reference 49 to obtain all the points;*** Participants received treatments 2 days a week.

HF: hot flash; NA: not available


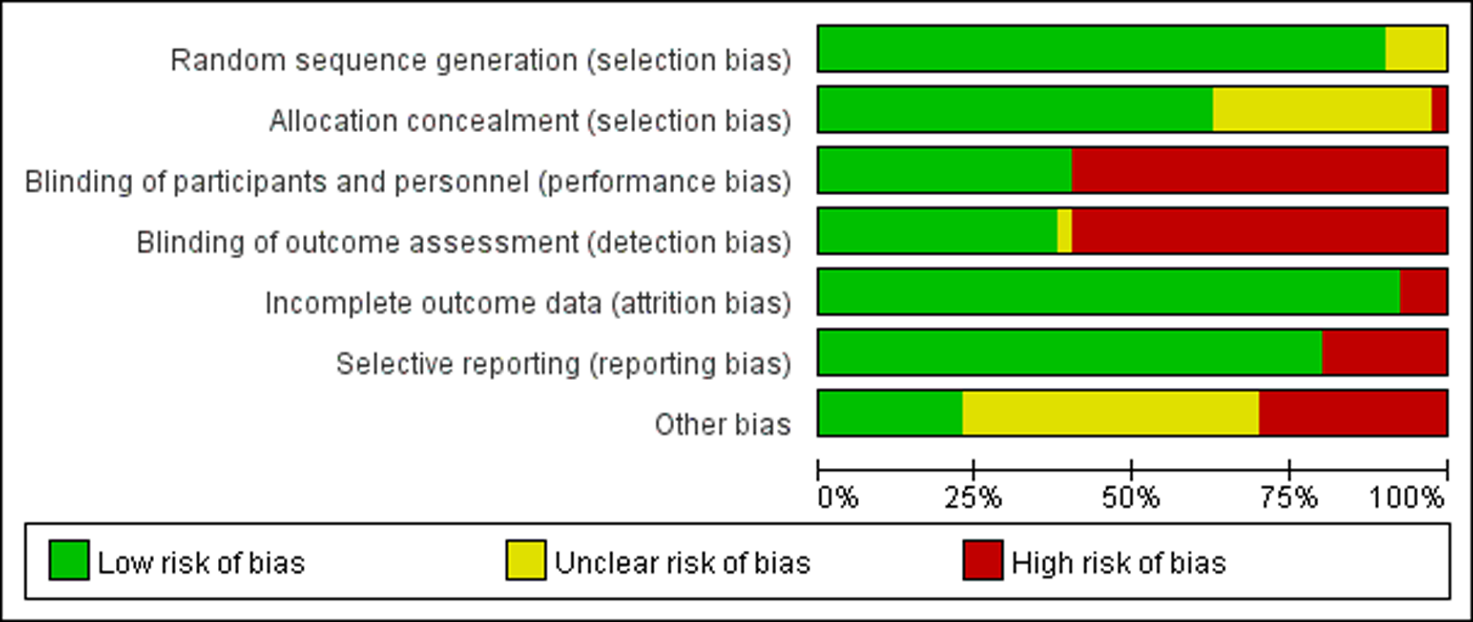


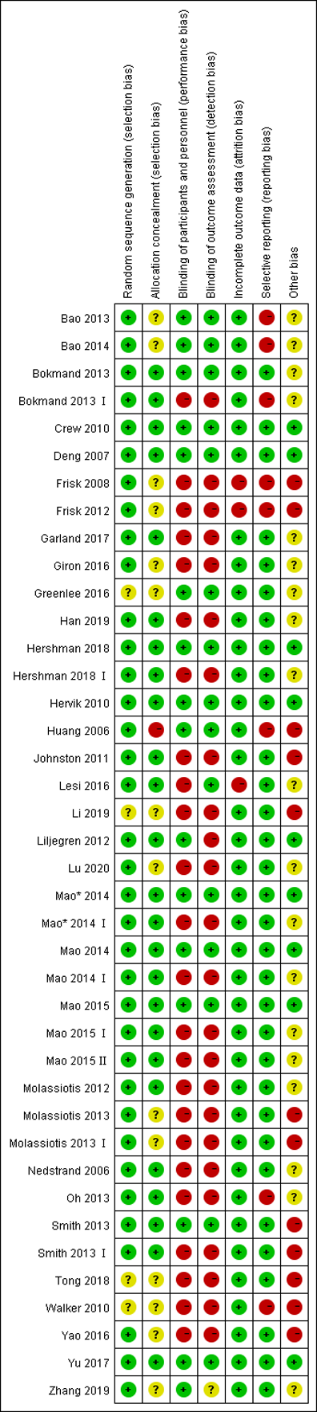

Supplement: Supplementary file 1 [file DataSheet_1.docx]
